# Slow Feature Analysis on Retinal Waves Leads to V1 Complex Cells

**DOI:** 10.1371/journal.pcbi.1003564

**Published:** 2014-05-08

**Authors:** Sven Dähne, Niko Wilbert, Laurenz Wiskott

**Affiliations:** 1 Machine Learning Group, Department of Computer Science, Berlin Institute of Technology, Berlin, Germany; 2 Institute for Theoretical Biology, Humboldt-University, Berlin, Germany; 3 Bernstein Center for Computational Neuroscience, Berlin, Germany; 4 Institute for Neural Computation, Ruhr-University Bochum, Bochum, Germany; University of Rochester, United States of America

## Abstract

The developing visual system of many mammalian species is partially structured and organized even before the onset of vision. Spontaneous neural activity, which spreads in waves across the retina, has been suggested to play a major role in these prenatal structuring processes. Recently, it has been shown that when employing an efficient coding strategy, such as sparse coding, these retinal activity patterns lead to basis functions that resemble optimal stimuli of simple cells in primary visual cortex (V1). Here we present the results of applying a coding strategy that optimizes for temporal slowness, namely Slow Feature Analysis (SFA), to a biologically plausible model of retinal waves. Previously, SFA has been successfully applied to model parts of the visual system, most notably in reproducing a rich set of complex-cell features by training SFA with quasi-natural image sequences. In the present work, we obtain SFA units that share a number of properties with cortical complex-cells by training on simulated retinal waves. The emergence of two distinct properties of the SFA units (phase invariance and orientation tuning) is thoroughly investigated via control experiments and mathematical analysis of the input-output functions found by SFA. The results support the idea that retinal waves share relevant temporal and spatial properties with natural visual input. Hence, retinal waves seem suitable training stimuli to learn invariances and thereby shape the developing early visual system such that it is best prepared for coding input from the natural world.

## Author Summary

It is believed that our sensory systems are adapted to statistical properties of behaviorally relevant elements in our natural environments. In the case of vision, one adaptation principle that has been put forward is the so-called slowness principle. However, the visual system is partially structured even before eye opening, when no natural input is available yet. Thus, spontaneous neural activity in the developing visual system of mammals (so-called retinal waves) has been suggested to contribute to shaping connections in early visual areas before the onset of vision. Here we aim to bring these two ideas together. Specifically, we apply an algorithm that implements the slowness principle to simulated retinal waves. The algorithm is set to encode the retinal wave input and thus has to extract relevant features from that input. After encoding, we are able to investigate the emerged representation and we find that the extracted features bear strong similarity to features that are encoded by neurons in the early visual system. These features are the building blocks for an object representation that is independent of the object's position in the visual field.

## Introduction

For us humans, vision is the most dominant sense. It is thus not surprising that the brain regions involved in visual processing, for example primary visual cortex (V1), have been subject to extensive investigation [1]. The conjunction of neuroscientific results and the study of statistical properties of natural images (i.e. the input to the visual system) have led to the idea that neurons found in V1 are well adapted to the statistical regularities present in natural images [2]–[5]. In fact, some of their response properties can be regarded near-optimal with respect to certain efficiency criteria [6]–[9]. So it seems that the design of early visual processing areas, i.e. the connectivity patterns between neurons in these areas, has evolved to cope best with images provided by the natural environment. These findings lead to an interesting question, namely how these well-designed connectivity patterns emerge as the visual system develops in the newly born (or even unborn) infant that has not yet been exposed to the natural environment.

Possible answers to this question parallel the debate of nature versus nurture. One position states that the connectivity patterns are stored in the genetic code while an opposing position advocates that the connectivity patterns are acquired once the organism is confronted with its natural input. In favor of the first position would be the fact that there are animals that can see right after birth [10] and that there are species that have a partially functioning visual cortex before eye-opening [11], [12]. In contrast to that, there are also studies that point out that the visual system needs visual input to fully develop its characteristic properties [13], [14] and is thus not likely to be fully determined by the genetic code. Most likely, the truth is to be found somewhere between these two positions.

One such combining approach states that the visual system indeed develops to optimize a certain objective function, but it does so prior to the onset of vision by learning on internally generated input. The learning objective and the input generating mechanism would have to be stored genetically while the actual wiring of the neuronal connections can be done dynamically, driven by the objective and the input statistics. This approach includes the possibility for further learning and adaption after the onset of vision in order to maximize the organism's adjustment to its environment. Thus, this *innate learning* approach [10] offers a possibility to combine the seemingly contradictory points of view about the development of the early visual system. In order to support the innate learning approach, two aspects have to be identified: what is the internally generated input and what is the learning objective.

A prime candidate for the internally generated input to the developing visual system are the so-called *retinal waves* [15]–[18]. The immature and yet light-insensitive retina of many animal species generates spontaneous bursting activity. This activity occurs in coherent spatiotemporal patterns that spread in waves across the retina and bring the spontaneous bursts of neighboring cells into synchrony. These wave-like activity patterns are not only present in the retina but in many other parts of the developing nervous system, such as the spinal cord, the hippocampus, the cochlea, and the cerebellum (see [18] for a review). The underlying mechanisms that give rise to the correlated bursting patterns are still subject of study. However, the important role of retinal waves in context of the development of the visual system is supported by theoretical as well as experimental studies [19], [20], suggesting that spatially correlated input is required for the proper development of ordered connections from the retina to the LGN and then to visual cortex [21]. Chemically abolishing retinal waves results in severe developmental impairment of cortical ocular dominance columns [11], [12] and orientation selectivity [13], [14], . Thus, retinal waves seem to be a necessary condition for the emergence of many important properties of the early visual system [16], [23].

As for the learning objective, there are two prominent candidates that have been applied in the context of computational modeling of the visual system, namely sparsity and slowness. Sparse coding, when trained with natural images, has been shown to lead to the emergence of functions that share relevant properties with specific types of V1 cells that are called simple cells [6] and complex cells [24], [25]. These types of objectives lead to computational algorithms that are called Independent Component Analysis (ICA). Recently, ICA has also been applied to retinal wave-like images, which results in simple cell-like functions and supports the innate learning mechanism hypothesis [10].

The second candidate objective is called temporal stability, or slowness [26]. In a number of studies, the slowness objective has been used to model the self-organized emergence of complex cells [8], [9], [27]. Additionally, the slowness principle has been successfully applied to model to emergence of hippocampal place cells [28]–[30] and for finding low dimensional representations of high dimensional sensory input that bear behavioral relevance [31], [32]. Slow Feature Analyis (SFA) [33] is an algorithm that efficiently implements the slowness objective by finding input-output functions that maximize an appropriate objective function on given training data. Note that while the slowness objective is indeed a biologically plausible coding objective, the SFA algorithm itself is not designed to be biologically plausible. SFA has found many applications in the field of computational neuroscience [9], [29], [31], [32] as well as time-series analysis and signal processing [34]–[36]. Furthermore, SFA has been studied analytically to a large extent and therefore predictions about the functions that SFA finds are possible, based on the statistics of the training data [37]–[40]. In [41] is was conjectured that SFA might be capable of finding complex cell-like functions when trained with retinal waves.

Thus, in this manuscript, we investigate to what extent it is possible to explain the emergence of complex cells based solely on two computational principles: spontaneous retinal wave activity and the slowness coding objective.

## Methods

### Slow feature analysis

The goal of SFA is to find instantaneous input-output functions *g*(**x**) that extract slowly varying scalar output signals from a high-dimensional input signal **x**. To ensure that the extracted output signals are informative, they are required to be uncorrelated and to have unit variance.

The optimization problem is mathematically formulated as follows. Given a multidimensional input signal 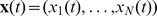, 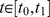, find a set of real-valued functions 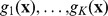 from a function space F, such that for the output signals 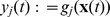 the expression
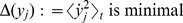
(1)under the constraints
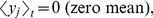
(2)

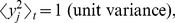
(3)

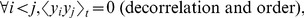
(4)with 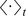 and 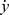 indicating time-averaging and the time derivative of *y*, respectively.

The expression to be minimized (1) is a measure of the temporal slowness of the signal *y_j_*(*t*), with small Δ-values indicating a slowly varying signal. The trivial solution is a set of functions that is constant for all *t*. Constraints (2) and (3) avoid this trivial solution and constraint (4) ensures that different functions code for different aspects of the input signal. The latter also introduces an order, *y*_1_ being the slowest signal, *y*_2_ the second slowest and so on. We refer the reader to [9], [33] for details on how this optimization is solved by means of SFA.

In the present application, we chose to perform SFA in the expanded input space of all polynomials up to a degree of two, which makes it possible to conveniently express the SFA solution in terms of a *quadratic form* of the original input signal,
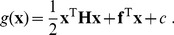
(5)The coefficients that constitute **H**, **f**, and *c* are determined by SFA and the constant *c* is subtracted to achieve zero mean.

In order to understand the response properties of *g*(**x**) to input stimuli, it is instructive to consider the eigenvalue decomposition of the matrix **H**. This decomposition is given by the sum over the outer products of its eigenvectors **v** *_i_*, weighted by the corresponding eigenvalues **λ** *_i_*:
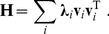
(6)In this formulation, the computation of the quadratic term becomes
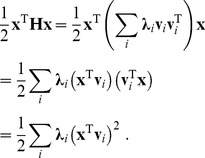
(7)Hence, the quadratic term can be regarded as a weighted sum of squared filter outputs to an input image, where the weights are given by the eigenvalues **λ** *_i_* and the corresponding filters are given by the eigenvectors **v** *_i_*.

SFA returns as many functions *g*(**x**) as there are dimensions in the expanded function space and the resulting SFA functions are ordered according to their temporal slowness, as measured on the training data. In the remainder of this article, we refer to each of the input-output functions *g*(**x**) as SFA units and limit our analysis to the 50 slowest varying functions.

#### Analysis of response properties

Since the trained SFA units are quadratic forms of the pixel intensities, there is an explicit formulation of the input-output relation. Given a fixed-norm constraint on the input images, it is therefore possible to compute the optimal excitatory and inhibitory stimuli [39], [40]. In addition, similar to physiological experiments, we compute the response of the SFA units to sinusoidal gratings. The orientation and phase of the gratings were confined to the range from 0 to 2*π*, whereas the maximal possible spatial frequency was eight cycles per receptive field, due to the 16 by 16 pixel size of the receptive field. For better comparison of the response properties between individual SFA units, the output of each unit was normalized in the following manner: First of all, the response of the unit to a gray input image was subtracted. Secondly, the output was sign corrected such that the maximal positive output is larger in magnitude than the maximal negative output. Finally, the output was normalized to have unit variance over the explored range of input stimulus parameters.

The full exploration of the parameter space allows us to extract for each unit the set of best parameter values, i.e. the constellation of spatial frequency, orientation and phase of the sinusoid that maximally excites the SFA unit. We visualize the units' responses as functions of the sinusoid parameters. In order to visualize these three dimensional response functions, we set one or two parameters to the values preferred by the SFA unit and plot the response as a function of the remaining parameters.

Additionally we employ two quantitative metrics that are used in physiological experiments as well: the *response modulation index* [42] (also referred to as F1/F0 ratio) and the *orientation selectivity index* (OSI) [13].

The F1/F0 ratio is a spectral measure of the phase-dependence of the response. It is the ratio of the first harmonic of the response to its DC component, hence the name F1/F0 ratio. Cortical cells with an F1/F0 ratio smaller than one are classified as complex cells, whereas cells with a ratio larger than one are classified as simple cells.

The OSI is a spectral measure of the orientation-dependence of the response and is given by
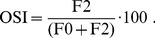
(8)Here F2 is the amplitude of the second harmonic of the orientation tuning curve while F0 is the average of the orientation tuning curve.

### Simulated retinal waves

In order to train the SFA units, we have used input image sequences that were derived from a biologically plausible model of retinal waves proposed by Godfrey and Swindale [43]. The retinal wave model was parameterized such that it produced waves that are similar in size and velocity to those observed in mice during the first two weeks after birth. The parameters were taken from [43].

The retinal wave model assumes that spontaneous activity of retinal amacrine cells drives the wave activity. In our simulations, the retinal amacrine cells were arranged in a regular grid consisting of 128 by 128 cells. Each cell received input from other cells that were within a six cell dendritic radius. All simulations were started with random membrane potentials and threshold levels for all cells. A cell was considered active as long as its membrane potential was above threshold. The activity of each cell was represented by a binary value and the simulated retina was visualized as a correspondingly sized image, black pixels represented non-active, white pixels represented active cells.

Such an arrangement leads to inhomogeneities at the borders of the simulated patch of retina, because those cells that are situated at the borders receive less input than those that are further away from the border. Godfrey and Swindale addressed this issue by means of position-dependent regulations of activation thresholds in order to achieve a uniform distribution of wave initiation points over the retina patch. They have also reported that using cyclic boundary conditions, i.e. connecting cells at opposite borders of the simulated retina, leads to the same result. In our implementation, we chose to use cyclic boundary conditions. In order to reduce noise in the input data, spatially not coherent activity was filtered out by setting those pixels to zero that did not have more than 4 active pixels among their 8 directly adjacent pixels.

**Figure 1:**
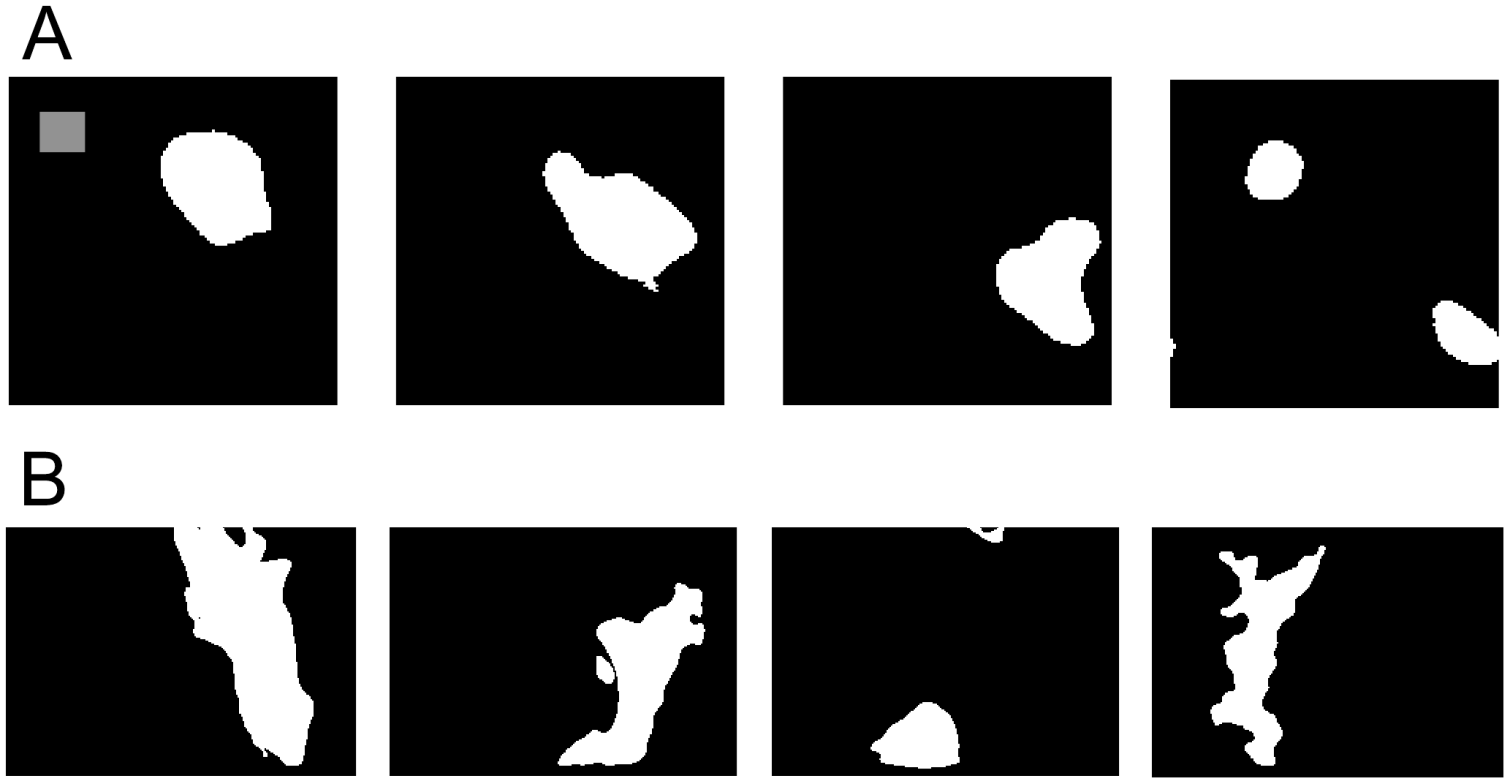
Simulated and real retinal waves. **A** Four example frames of simulated amacrine cell activity with a time distance of 20 frames (i.e. 5 seconds simulated time). Receptive field size is illustrated by the gray square in the lop left corner of the first frame. **B** Calcium imaging plots of a patch of mouse retina, adapted from Toychiev et al. (2013) [44], by means of smoothing and thresholding to produce a binary image of the activity.

After an initial warm-up phase of 30 minutes simulated time, the produced retinal activity patterns were recorded for another 15 min simulated time, which resulted in 3600 binary images (4 per second). Figure 1 A shows four sample frames of the full simulated retina. In order to illustrate the dynamics of the waves, the images in the example sequence are each 20 simulated time steps apart, which corresponds to 5 seconds. So the four depicted images show 15 seconds of simulated activity. Figure 1 B show experimental results for comparison [44].

Simulating the patch of retina is costly in terms of computational time. However, due to the cyclic boundary conditions and the otherwise homogeneous structure of the simulated retina patch, we can make an ergodicity assumption here and combine the information from several spatial locations. The image sequence obtained was tiled into overlapping receptive fields of size 16 by 16 pixels. The overlap between receptive fields was 5 pixel, resulting in 289 image sequences, each being 3600 images long and having a dimension of 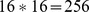. The 289 receptive field image sequences were concatenated and separator frames (containing only zeros) were inserted between the individual receptive field image sequences. These frame separators avoid jumps of activity in the training signal. The concatenated image sequences contained 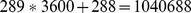 images, yielding a total of 4336 minutes (approx. 72 hours) simulated time.

Prior to the quadratic expansion of the input data, the dimensionality of the concatenated image sequence was reduced to 50 by applying principal component analysis (PCA). This greatly reduces the computational costs of the SFA algorithm in terms of memory requirements. We elaborate on the effects of dimensionality reduction by means of PCA in the Discussion section. Finally, the concatenated and dimensionality reduced image sequence served as input to SFA.

## Results

The trained SFA units are characterized by their optimal stimuli and their tuning to sinusoidal gratings.

### Optimal stimuli resemble Gabor patches

**Figure 2:**
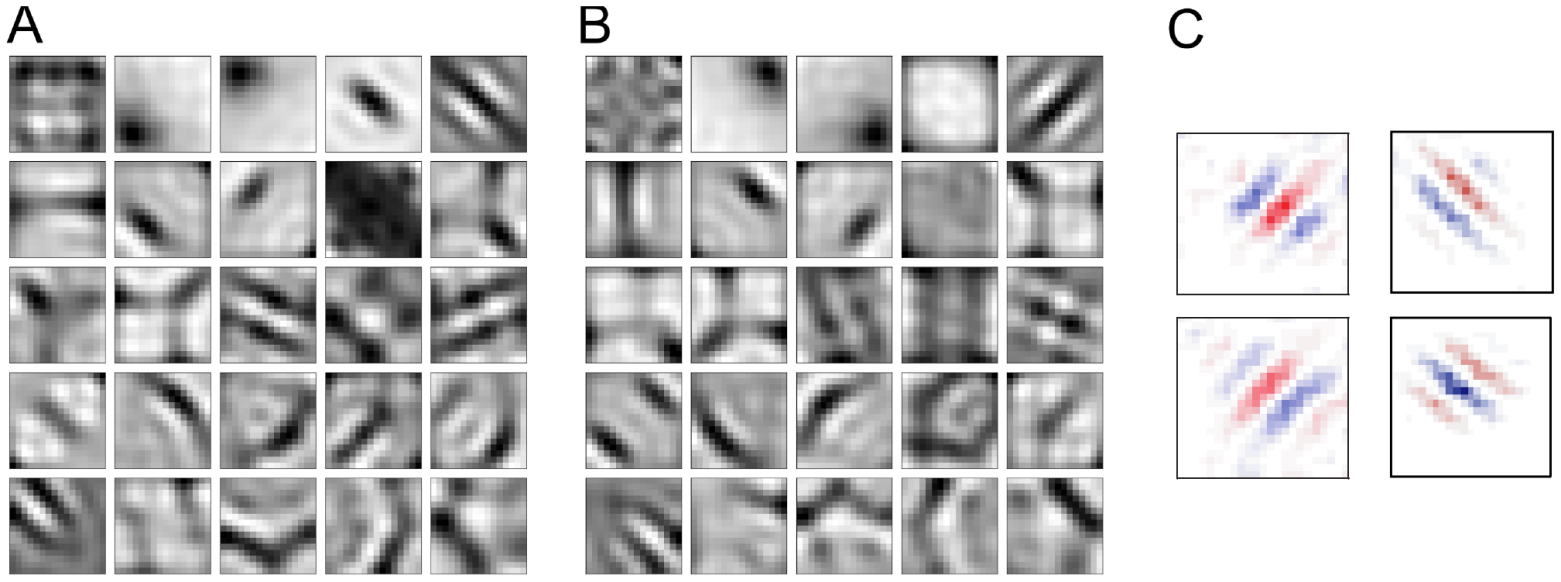
Optimal stimuli. **A** Maximally excitatory stimuli, plotted for the first 25 SFA units. **B** Maximally inhibitory stimuli for the same selection of units as in **A**. **C** For comparison, optimal stimuli of complex cells of mature cats with normal visual experience estimated by Felsen et al. (2005) [50].

Once the structure of a cortical cell's optimal stimulus has been estimated in neurophysiological experiments, it allows inferences about the preferred orientation, frequency, and (in case of simple cells) the preferred phase of the cell. Simple cell optimal stimuli can be mapped by computing the spike-triggered average of random dot input stimuli and are well described by 2D Gabor functions [45]–[47]. Complex cells, on the other hand, require more elaborate schemes for finding optimal stimuli, due to their largely non-linear input-output relation. Using methods such as spike-triggered covariance or second order interaction maps has revealed many insights about the spatial structure of complex cell optimal stimuli [48]–[51]. Additionally, stimulus optimization can be employed for finding effective stimuli that elicit response from sensory cells [52], [53]. For example, just like those of simple cells, the optimal stimuli of complex cells also posses subregions with opposite polarity (similar to ON and OFF regions), from which the frequency tuning of the cell can be predicted. Figure 2 A shows the maximally excitatory stimuli for the first 25 SFA units (i.e. the 25 slowest), whereas Figure 2 B shows the maximally inhibitory stimuli for the same units. Most of the optimal stimuli (excitatory as well as inhibitory) show spatially segregated and elongated ON and OFF regions, which is in close correspondence with experimental data. See Figure 2 C for a comparison with optimal stimuli obtained from adult cats with normal visual experience, reported in [50].

### Responses to sinusoidal gratings reveal phase invariance and orientation selectivity

Further response properties of the SFA units are visualized by showing their responses to sinusoidal gratings. When V1 cells are probed with sinusoidal gratings in neurophysiological experiments, the used gratings are usually parameterized along three dimensions: orientation, spatial frequency, and phase. Here we adopt the same convention and parameterize the input gratings accordingly.

**Figure 3:**
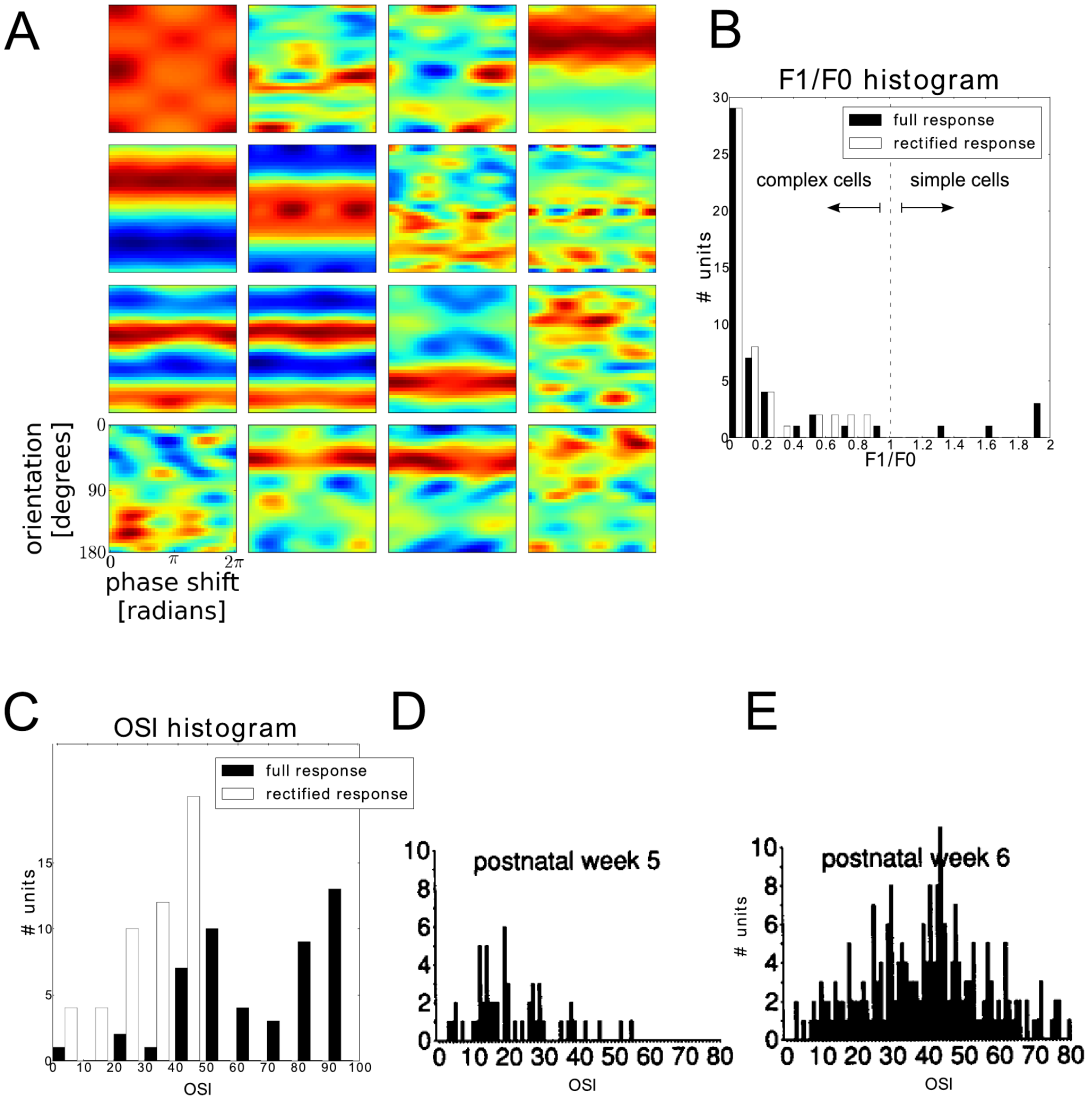
Response of the SFA units with respect to phase and orientation of sinusoidal gratings. **A** Responses to sinusoidal gratings depicted as a function of orientation (y-axis) and phase (x-axis) of the grating. The spatial frequency was set to the unit's preferred value. **B** Histogram of response modulation (F1/F0) values, indicating the susceptibility to the phase parameter of the input grating. Black bars correspond to using the full response of the units to compute F1/F0 values, white bars correspond to using the half-wave rectified response. This histogram is illustrates that most of the SFA units from this simulation run (45 out of 50) have an F1/F0 value smaller than one and would thus be classified as complex cells in a physiological experiment. It cannot be expected to resemble the distribution of simple and complex cells in visual cortex. **C** Histogram of orientation selectivity (OSI) values, indicating the susceptibility to the orientation parameter of the input grating. Black bars correspond to using the full response of the units to compute OSI values, white bars correspond to using the half-wave rectified response. **D** and **E** show OSI histograms obtained from ferrets (adapted with permission from Figure 2 of Chapman B et al. (1993) [13]). Eye opening occurs between postnatal week 5 and 6. See last paragraphs of the Results section for a thorough investigation of the reasons for high OSI values.

Figure 3 depicts the responses of the first 25 SFA units as a function of orientation and phase of the input sinusoidal grating, which we refer to as *orientation/phase-dependent response*. The spatial frequency of the gratings was set to the value that maximizes the response of the respective SFA unit. Most of the orientation/phase-dependent responses exhibit horizontal stripe patterns, indicating that the response varies stronger along the orientation axis compared to the phase axis.

This rather qualitative observation was quantified using the response modulation index (F1/F0 ratio) and the orientation selectivity index (OSI). Figure 3 B and C show the corresponding histograms of these two measures, computed for the 50 SFA units of this particular simulation run. It is obvious that taking into account the full inhibition in the simulated units in the OSI and the F1/F0 ratio can lead to unrealistic values, given that physiological units cannot have negative firing rates but can at most have suppressed firing below spontaneous firing rate. We therefore also show histograms of the half-wave rectified unit activities. Depending on the level of spontaneous activity, the most plausible histograms for the simulated units will lie somewhere between the raw and the rectified histogram.

The majority of SFA units have a response modulation index smaller than one. Hence, they would be classified as complex cells in a physiological experiment. The distribution of orientation selectivity values shows a wide spread over the possible range of values, which is consistent with experimental findings [13]. However, the comparatively high average value (68.7) indicates a rather specific orientation tuning in the majority of SFA units. Some units even reach OSI values above 90, which is much higher than the values reported for adult cats or ferrets. OSI histograms for ferrets shortly before eye-opening and shortly afterwards are shown in Figure 3 D and E, respectively. How exactly SFA achieves the phase invariance and the large orientation selectivity is an interesting issue and considered in more detail later in this section.

**Figure 4:**
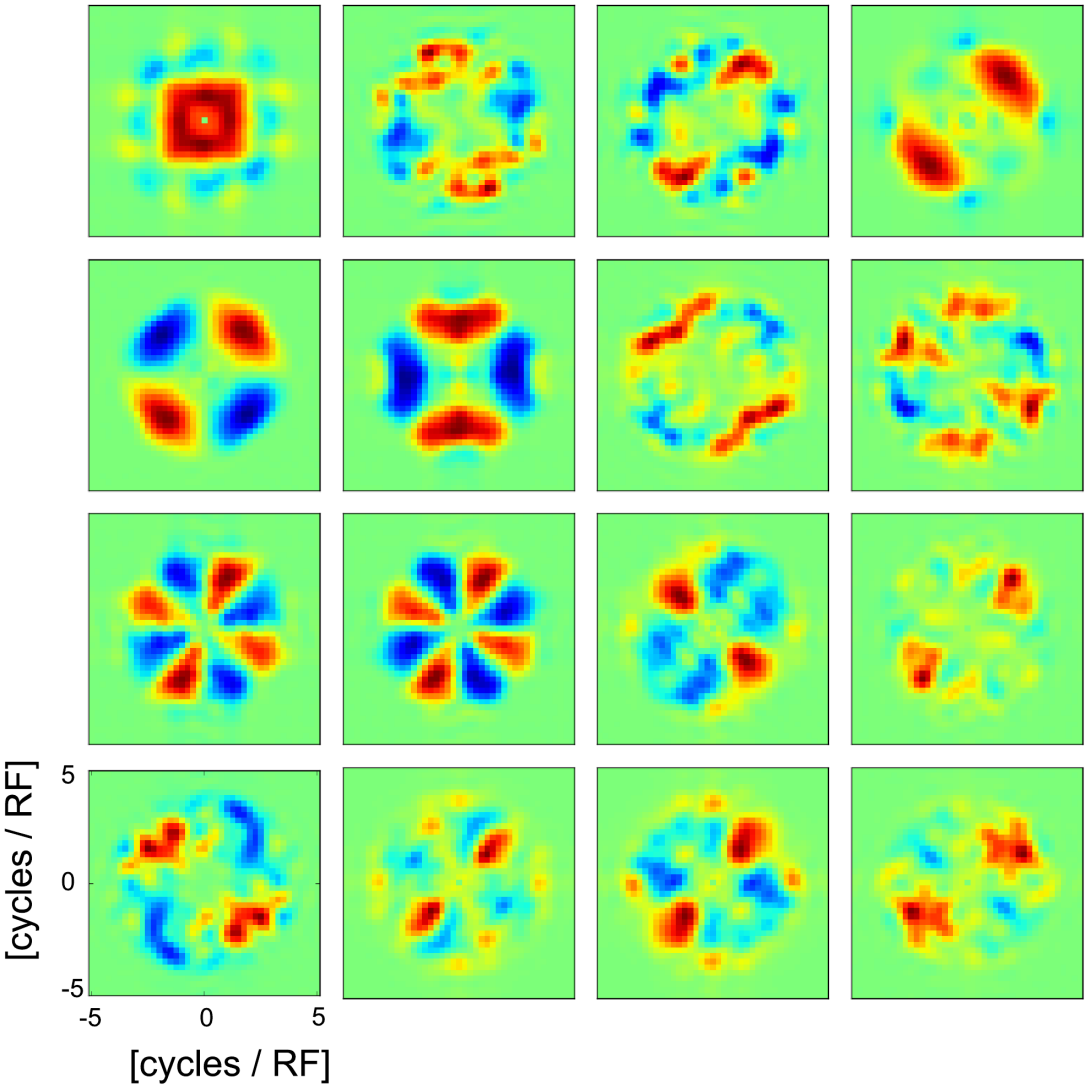
Response of the SFA units as a function of spatial frequency of sinusoidal gratings. Response of the SFA units to sinusoidal gratings depicted as a function of the gratings' spatial frequency and orientation. Warm (cold) colors indicate excitatory (inhibitory) response. The results are qualitatively similar to those obtained for V1 neurons in macaque monkeys (see for example Figure 2 in Ringach et al. (2002) [54]).

Figure 4 shows the response of the SFA units as a function of spatial frequency (radial direction) and orientation (azimut) of the input gratings (averaged over phase). Ringach et al. [54] have investigated the response of V1 cells in macaque monkeys in the same manner. Similar to Ringach et al.'s findings, almost all SFA units exhibit active inhibition to stimuli that are not oriented in a preferred direction. This inhibition takes place for orientations orthogonal to the unit's preferred orientation but in some units also for non-orthogonal directions.

**Figure 5:**
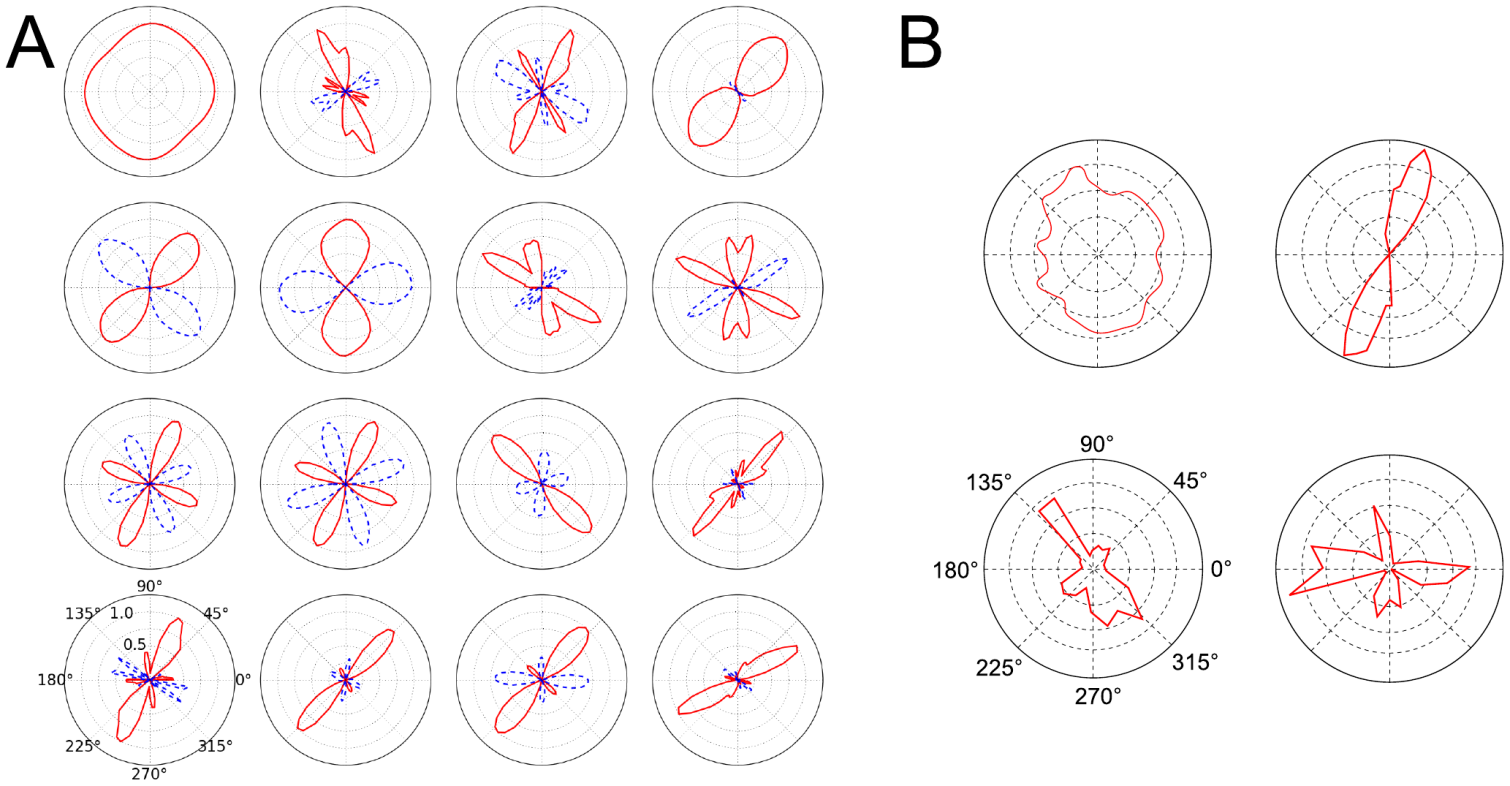
Response of the SFA units as a function of orientation of sinusoidal gratings. **A** SFA unit response as a function of orientation. Solid red (dashed blue) lines indicate excitatory (inhibitory) response. **B** Orientation tuning functions of V1 cells of macaque monkey (figure adapted from DeValois et al. (1982) [55]). Note that inhibitory effects were not investigated by DeValois et al. [55], and thus there are no inhibitory responses plotted in **B**.

The orthogonal and non-orthogonal suppression is also visible in typical orientation tuning polar plots, depicted in Figure 5 A for the same SFA units that are shown in Figure 4. These plots show the orientation tuning function at the unit's preferred frequency and phase. Unlike traditional plots of this kind, here also the negative response (inhibition) of the SFA units is shown. Excitatory activity is plotted in solid red lines, while inhibition is plotted in dashed blue lines. The majority of SFA units show a clear orientation preference. There are units that prefer only a single orientation as well as units that also respond strongly to a second direction, which is in some cases orthogonal to the first one and in other cases not. This phenomenon manifests itself in the polar plots as so-called secondary response lobes within the excitatory (inhibitory) response curve. Such secondary response lobes are also observed in cells found in mammalian V1. Figure 5 B shows experimental data obtained by [55]. Here only the excitatory response is plotted, but the different types of responses (not tuned for orientation, single orientation preference, secondary response lobes) are well exemplified (compare with solid red lines in Figure 5 A). The inhibitory response of the SFA units seems to follow similar patterns as the excitatory response. There is often inhibition in a single direction only but also secondary inhibition response lobes are seen.

### Phase invariance through emergence of Gabor quadrature filter pairs

Most of the SFA units show a large invariance in their response with respect to phase (or position) of an input grating. Here we address the question how the SFA units achieve the phase invariance.

In the classical complex cell model [56] this phase invariance is a direct result of pooling the squared outputs of two linear Gabor filters that have the same preferred frequency and orientation but are in 90° phase shift relative to each other. Such a pair of filters is called a *quadrature filter pair* (QFP). Recall that the SFA units are quadratic forms of the pixel intensities, in which the contribution of the quadratic and the linear term can be separately investigated (see equation 5). The constant term in the quadratic form in equation 5 is of no interest in the analysis, because it cannot convey any information about a changing stimulus. The linear term of the quadratic form alone cannot achieve phase invariance either. However, the quadratic term as well as the linear term in conjunction with the quadratic term can in principle achieve phase invariance. Thus it is those two terms that we analyze further.

**Figure 6:**
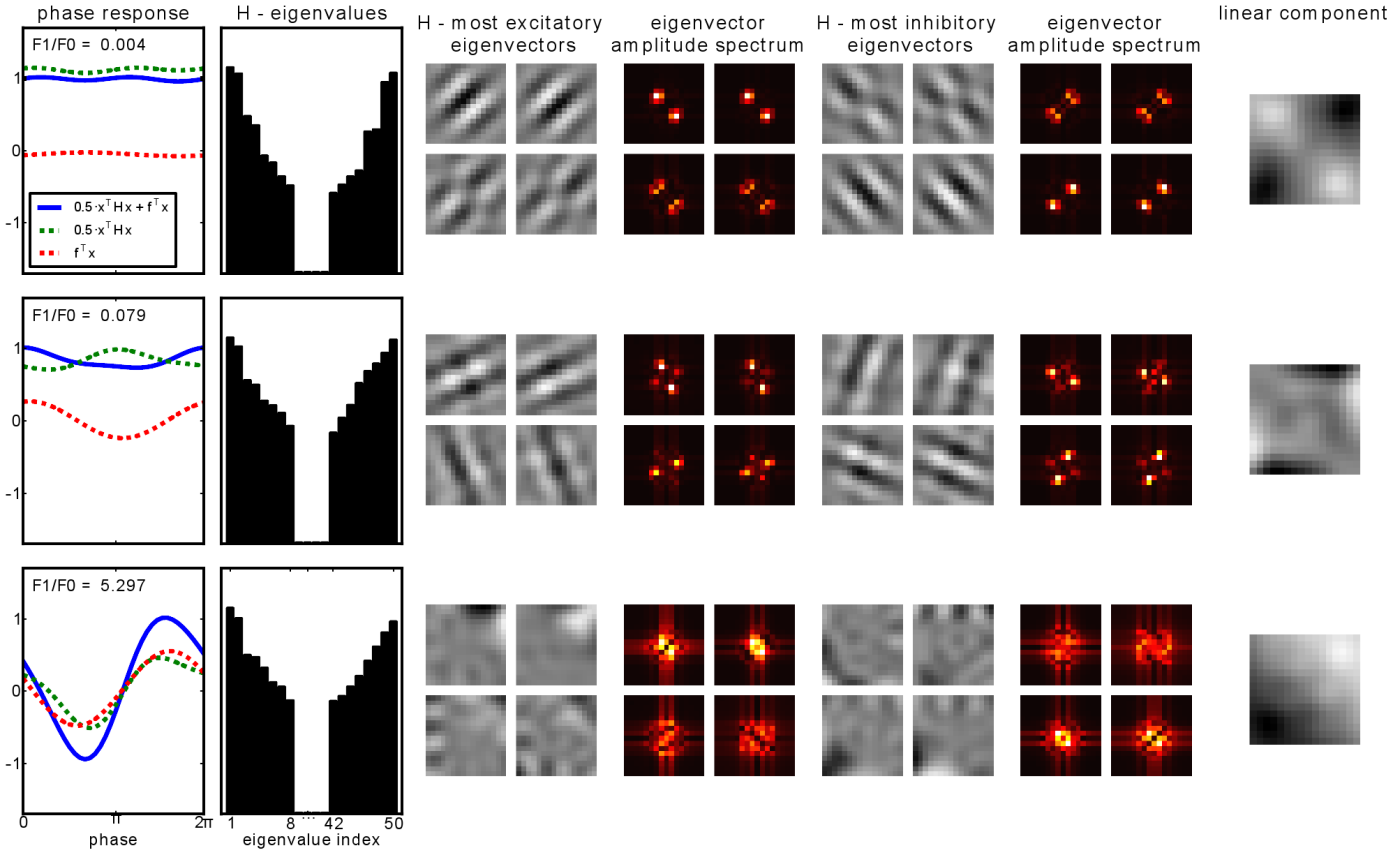
Emergence of phase invariance. The two top rows correspond to the most phase invariant SFA units, the bottom row to a unit whose response is phase dependent. Phase dependence of the linear and the quadratic term as well as their sum is depicted in the first column. The second column shows the two ends of the eigenvalue spectrum of **H**. Eigenvalues are sorted from highest to lowest, then their absolute values are plotted. Columns three and five show the eigenvectors that belong to the four largest, respectively smallest, eigenvalues, plotted as images. Columns four and six show the corresponding Fourier amplitude spectra. The last column shows the linear component of the quadratic form plotted as an image.

In the first column of Figure 6, we show the output of the quadratic term, the output of the linear, and their sum as a function of phase of the input grating for a selection of SFA units. Spatial frequency and orientation of the input gratings have been set to the units' preferred values. The SFA units chosen include the two that exhibit the largest phase invariance (smallest F1/F0 ratio) and the one that exhibits the smallest phase invariance (largest F1/F0 ratio), shown in the two top rows and bottom row, respectively. For the most phase invariant unit, it can be seen that the phase invariant response is mostly carried by the contribution of the quadratic term. The contribution of the linear term on the other hand is marginal in this case. For the second unit (middle row in the same figure) the response of the linear term is larger but seems to cancel the phase dependent fluctuations of the quadratic term, which are stronger compared to the unit above. However, for the unit with the lowest phase invariance, both terms show an almost aligned phase dependence and contribute almost equally to the overall output, which leads to a very high F1/F0 ratio for *g*(**x**), i.e. small phase invariance. Figure 6 shows the largest-magnitude eigenvalues and the corresponding eigenvectors of **H** for the selected SFA units, plotted as images, as well as corresponding amplitude spectra. The last column in the figure shows the linear component.

For the most phase invariant unit (top row in the figure), the largest-magnitude eigenvectors seem to come in pairs of two. Additionally, the corresponding eigenvectors are Gabor filters with the same orientation and spatial frequency, but with a 90° phase shift. Thus, they form a quadrature filter pair. The squaring of their respective outputs and the weighting with approximately the same factor (i.e. their eigenvalues) leads to a phase invariant response when stimulated with a grating of preferred orientation and spatial frequency, as was the case here. The preferred orientation of this SFA unit is that of the eigenvector corresponding to the largest eigenvalue. Interestingly, if the orientation of the test grating deviates somewhat, the response of that unit is still large, because of the contribution of the third and fourth eigenvectors. Their preferred orientation is similar (yet wider) compared to the first and second eigenvector. This leads to a broad orientation tuning of this particular SFA unit.

The second-most phase invariant SFA unit (middle row) also shows eigenvectors of **H** that resemble Gabor filters that come pairwise and in 90° phase shift. However, the spectrum of eigenvalues does not show as many pairwise occurring eigenvalues as was the case in the most phase invariant unit. Thus, the squared output of the corresponding filters in the quadrature filter pairs are not as equally weighted and thereby leads to oscillations in the output of 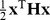. Here the orientation of the third and fourth eigenvector is orthogonal to that of the first and second and thus leads to secondary response lobes in the orientation-dependent response curve of this SFA unit.

The eigenvectors of the third unit shown (bottom row) do not resemble phase shifted pairs of Gabor filters. The lack of appropriate filters and corresponding pairwise eigenvalues leads to strong oscillations in the phase dependent response curve, i.e. very little phase invariance. Such a cell may, however, be very invariant to other features (orientation, contrast, etc.), which were not varied in this particular test.

To summarize, we have found that the phase invariance of the SFA units is explained by two features, namely (1) the emergence of pairs of Gabor filters with equal orientation and spatial frequency but 90° phase shift, and (2) the emergence of correspondingly pairwise eigenvalues, which together constitute Gabor quadrature filter pairs. This property is observed in about half of the SFA units obtained.

### Large OSI values arise due to smooth orientation tuning curves and inhibitory responses

The histogram of OSI values in Figure 3 shows that a large number of SFA units seem to posses rather high orientation selectivity values. OSI values of 90 and higher are, to the best of our knowledge, not reached by actual cortical neurons. Values of up to 80 (and equivalent values in other scales) are possible, yet very rare [13], [14]. Here we investigate the reasons for the large OSIs of SFA units.

Recall that the OSI measures the ratio between the second harmonic of the orientation tuning curve (F2) and the sum of F2 and the average response across all orientations (F0). Thus two scenarios can lead to large a OSI: Firstly, the OSI becomes large if there are two peaks in the orientation tuning that are separated by 180° and the response to other directions is comparably low. Secondly, the OSI becomes large if the average response (F0) is close to zero. In fact, the OSI is maximal if F0 equals zero, independent of how orientation selective the analysed SFA unit actually is.

In the SFA units obtained, both effects work together and thereby yield large OSI values. The presence of rotation in the training data is predicted to lead to harmonic oscillations in the orientation tuning curve [37], [41]. When visualizing such orientation tuning in polar plots (see Figure 3), the harmonic oscillation is manifested via inhibitory lobes with amplitudes comparable to the excitatory lobes. The presence of secondary (and possibly more) excitatory and inhibitory lobes in the polar plots represent harmonic oscillations of higher frequency. These harmonic oscillations in the orientation tuning lead to smooth response curves with minimal average orientation tuning and thereby to large OSIs.

### Are smooth orientation tuning curves of SFA units due to detectable rotation in the training data or merely an artifact of the receptive field size?

Interestingly, there is another mechanism that could lead to smooth orientation tuning curves of SFA units, namely the combination of (i) finite receptive field size and (ii) input dynamics that are dominated by translation. In the case where the training input contains only translation and no rotation at all, the theory behind SFA predicts a rather erratic orientation tuning [41]. In the extreme case of infinitely large receptive fields, the translation in the input leads to the emergence of infinitely large quadrature filter pairs. This in turn corresponds to infinitely sharp peaks in Fourier space. The predicted orientation tuning curve would then be a weighted sum of such sharp peaks and thus not be expected to be smooth at all. However, due to the finite size of spatial receptive fields, the localization in Fourier space becomes less sharp, leading to a smoothing of the orientation tuning curve. Hence, even if trained with stimuli that do not contain rotation, a smooth, i.e. somewhat slowly varying, orientation tuning curve can still be expected, with the smoothness not emerging due to the slowness objective but as an artifact of the finite size of the receptive field. Thus the question arises whether the smooth orientation tuning curves of the SFA units obtained are due to detectable rotation in the training data or merely an artifact of the receptive field size.

**Figure 7:**
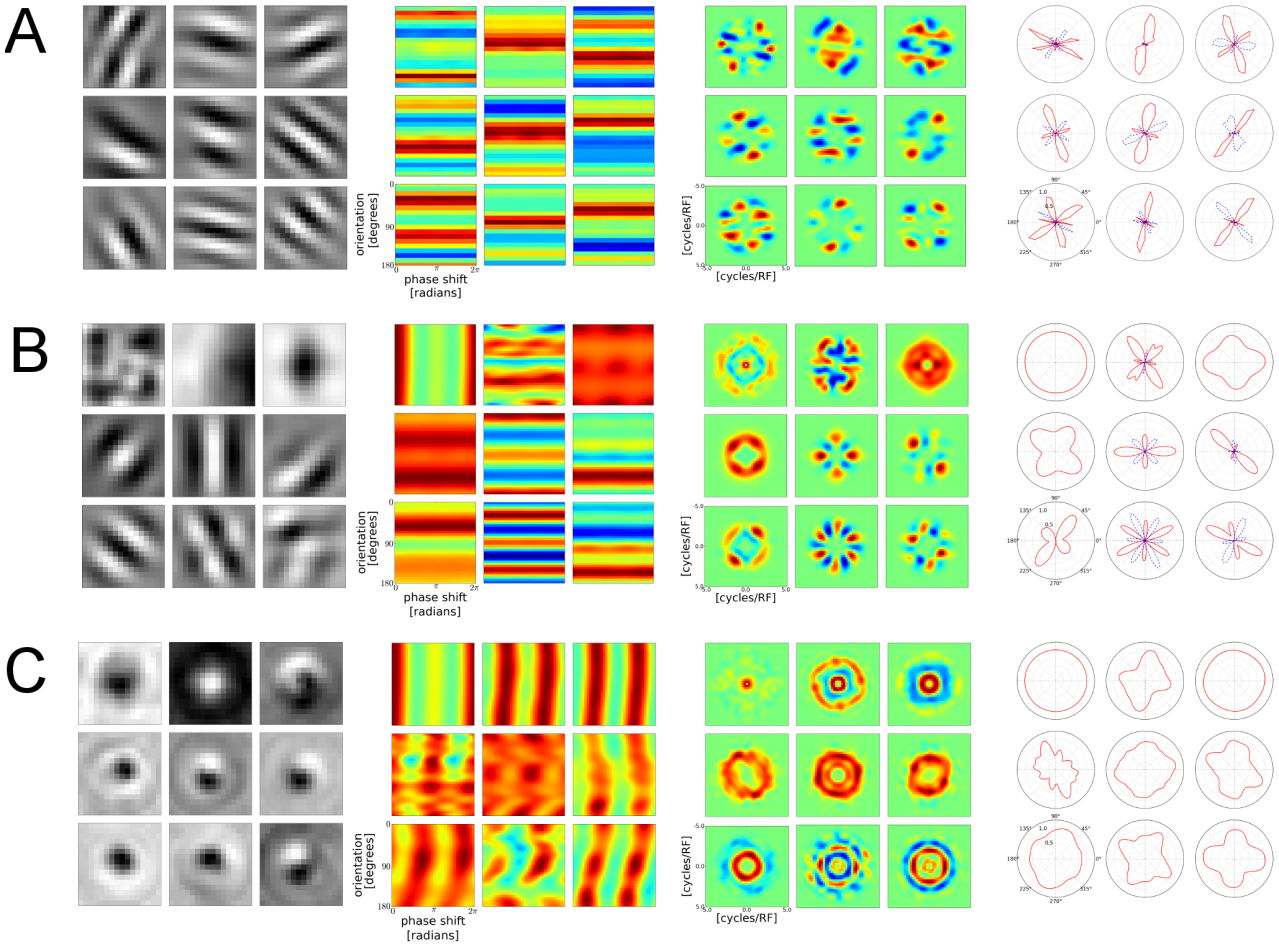
Translation versus rotation as dominant training input feature. Every plot group visualizes a different aspect of the trained SFA units and within each plot group, nine units are shown (every third, starting from the first, up to SFA unit number 25). Columns from left to right: optimal excitatory stimuli, orientation/phase response, response in Fourier space, orientation tuning plots. Rows: **A** SFA units trained with pink noise images that were subject to translation only. **B** Units trained with a mixture of rotation and translation. **C** Units trained with input that contained rotation only.

In order to answer this question we have conducted additional control experiments. Specifically, we conducted simulations in which SFA units were trained with pink noise image sequences that contained either only translation, only rotation, or a combination of translation and rotation applied to the images. A similar simulation protocol was used in [9]. Properties of the resulting SFA units are presented in Figure 7. In that figure we plot the same properties of SFA units that were shown in earlier sections for SFA units trained on simulated retinal waves.

**Figure 8:**
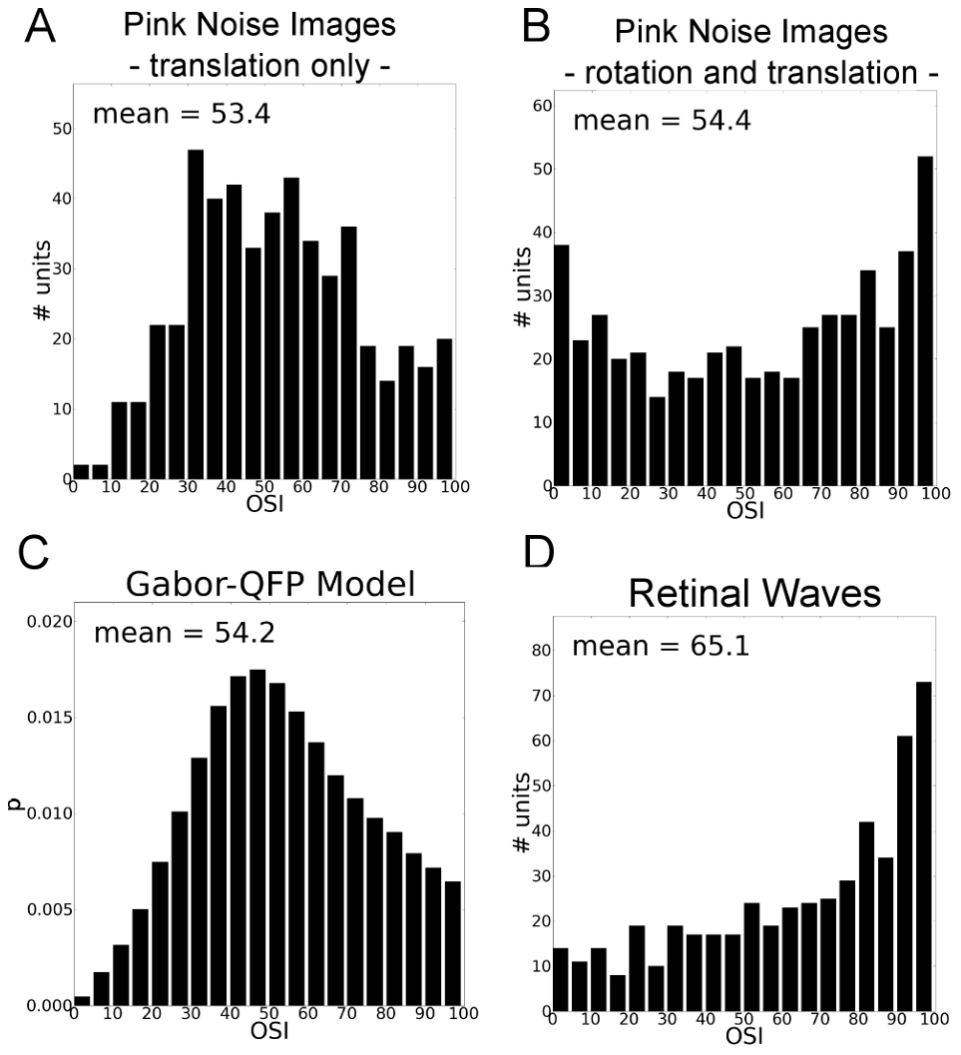
Orientation selectivity index (OSI) histograms of SFA units trained with different types of stimuli. **A** Training with pink noise images that were translated only. **B** Training with pink noise images that were rotated and translated. **C** OSI distribution of the Gabor-QFP model. **D** Training with retinal wave image sequences. Histograms in **A**, **B**, and **D** were obtained by pooling OSI values from the first 50 SFA units of 10 simulation runs with identical parameters for the training input generation.

Additionally, we analytically derived the OSI for a complex cell model that consists of a number of Gabor patch quadrature filter pairs (Gabor-QFP model), see text S1. In Figure 8 we show the OSI histograms of SFA units obtained from control experiments in which the input either contained translation only or translation as well as rotation. Furthermore, the figure shows the OSI histogram of the Gabor-QFP model and the OSI histogram of SFA units trained on simulated retinal waves. The OSI histograms of translation-only SFA units bears strong similarity to the Gabor-QFP model OSI histogram, yet both histograms exhibit a qualitatively different shape than that of retinal wave SFA units.

From this we conclude that the smoothly varying orientation tuning curves we obtained from retinal waves are not an artifact of finite receptive field size. Instead, the orientation tuning is a direct result of the SFA units adapting to rotational components in the input data.

## Discussion

In this article, we present the results of applying slow feature analysis to image sequences derived from a model of retinal waves. The resulting SFA units share a number of properties with complex cells, which are found in adult mammalian primary visual cortex. The defining feature of cortical complex cells is that they respond well to sinusoidal gratings and show little variation in their response when the phase of the grating changes. This most important feature can be reproduced with the SFA model, i.e. the response of the SFA units is largely invariant with respect to the phase of a sinusoidal input grating. Secondly, the optimal stimuli of a large portion of the SFA units show structure similar to that found in experimentally observed receptive fields. Many optimal stimuli resemble Gabor patches. Thirdly, similar to cortical simple and complex cells, the SFA units respond stronger to some orientations of the input grating than to others, i.e. they exhibit an orientation tuning. However, some specifics of their orientation tuning are not in accordance with physiological observations (see Result section and below for further discussion of this issue). Finally, the SFA units learned exhibit some degree of frequency tuning, which is also found to be the case in cortical simple and complex cells.

### Relation to other studies

Previously, models based on temporal slowness have been applied to natural image sequences and have reproduced properties that are reminiscent of cortical complex cells [8], [9], [27]. Similar results have been obtained with models based on bilinear sparse coding [25] or topographic ICA [24]. Yet none of these studies can answer the question whether retinal waves are sufficient stimuli to cause the development of complex cells. However, given that our results on retinal waves are comparable to those achieved on natural image sequences with a similar SFA model, we predict that complex cell properties should also arise when training the afore mentioned models with retinal wave image sequences.

Albert et al. have applied sparse coding [6] to static retinal wave images, which resulted in the emergence of basis functions having receptive fields similar to those found for cortical simple cells. The authors propose that the early visual system is structured under the same learning objective before and during visual experience. For retinal waves to be adequate training stimuli under a fixed objective, the waves must share relevant statistical properties with input acquired after the onset of vision, i.e. natural image sequences. Their results show that retinal waves share relevant spatial statistics with natural images and thus led to the emergence of simple cell receptive fields. However, for the slowness objective it is temporal statistics in the training data that is of great importance. The temporal statistics of an image sequence are governed by the spatial statistics and the type of image transformation that lead from one image to the next. Thus there is an intimate relationship between temporal and spatial statistics of image sequences. The results presented in our article indicate that retinal waves share relevant temporal statistical properties with natural visual input that suffice to induce the emergence of complex cell-like coding under the slowness objective.

### Biological plausibility of the retinal wave model

The retinal wave image sequences used for training the SFA units were derived from a model of retinal waves proposed by Godfrey and Swindale [43]. Their model explains the emergence of spatially coherent patterns and their propagation on the basis of spontaneous depolarization and activity dependent refractoriness of amacrine cells. Other models of retinal waves exist, see for example [57]–[60]. Despite the (sometimes subtle) differences in the wave generation mechanisms, all of the models mentioned reproduce relevant retinal wave characteristics such as the distribution of size, speed, and inter-wave-interval, as well as the spatial coherence and the spatially limited and changing wave domains. The Godfrey and Swindale model was chosen for practical reasons including the fact that their model was the most recent when the work in this project began, their model is easily implemented, it runs fairly quickly, and the authors included parameter settings for retinal waves of several animal species. However, there is no principal reason for favoring the Godfrey and Swindale model over the others. The results obtained should not be much different when using a different retinal wave model, provided that the model generates waves with similar statistics.

### Effects of PCA preprocessing of input data

Recall that we have applied PCA dimensionality reduction to the simulated retinal wave image sequences before using them as input to SFA. Dimensionality reduction was a necessary step for reasons of computational efficiency. SFA acts on second moment matrices that have to be estimated on the quadratically expanded input data. For 256 input dimensions, the number of entries of these matrices is in the order of 10^9^, while for 50 input dimensions the number of entries to estimate is in the order of 10^6^.

Applying PCA for dimensionality reduction is essentially equivalent to spatially low-pass filtering the input, due to the characteristic fall-off of power in the Fourier spectrum of natural images, as well as the spectrum of simulated retinal waves (see above for a discussion of the spatial statistics). Yet the dimensionality reduction allows to use larger receptive fields compared to using unprocessed input. Larger receptive fields in turn allow for an easier analysis, especially with respect to the interpretation of optimal stimuli. Berkes and Wiskott have thoroughly discussed the effects of PCA dimensionality reduction prior to SFA in [9], section 5.3 and Appendix A.1 as well as A.4. The main result of their analysis is that there is no qualitative difference between using large low-pass filtered patches and using smaller unprocessed patches.

However, using PCA processed (or low-pass filtered) image patches limits the extent of frequency-specific response of any subsequent coding mechanism. Similar to Berkes and Wiskott [9] we used 50 PCA components, which imposes a spatial frequency cut-off of about 4 cycles per receptive field, which is comparable to the cut-offs used in [6], [61]. See also [62] for the low-pass filter properties of the retina. Thus, after PCA processing or spatially low-pass filtering it is expected that there is no response to sinusoidal gratings with frequencies above the corresponding frequency cut-off. However, the frequency-specific response observed below this cut-off is independent of the PCA preprocessing and can thus only be attributed to the SFA itself. We find that many SFA units show frequency specific responses in the range of 0 to 4 cycles per receptive field (see Figure 4 A) despite the fact that they act on the exact same PCA-processed input.

### Biological plausibility of SFA

The SFA algorithm as such is not intended to be biologically plausible in any detail. It is an abstract implementation of the slowness principle. Thus SFA may produce effects that an actual neural system would not, or it may fail to reproduce certain aspects of actual neural system responses. However, SFA is a self-organizing learning algorithm which implements the slowness principle and thus it is expected to reproduce properties of neural systems that are directly related to the coding objective. In the present application, that particular property is phase invariance. The phase invariance of cortical complex cells can be well reproduced, which renders SFA a suitable model for the emergence of such. Furthermore, the SFA units obtained show direction- and frequency preference as well as secondary response lobes. In addition to the properties reproduced here, the modeling study of Berkes and Wiskott [9] has shown that SFA can also account for additional complex-cell features such as end- and side-inhibition.

Note that the standard complex cell model that consists of a single quadrature filter pair only is unable to produce response properties such as secondary response lobes or end- and side-inhibition. More than one pair is necessary to achieve these properties, which indicates that while the standard single quadrature pair model is sufficient to account for phase invariance and orientation selectivity, it is too simple a model to account for the properties previously mentioned. The SFA model, on the other hand, is complex enough to enable the emergence of multiple quadrature filter pairs.

The observed emergence of multiple quadrature filter pairs is in line with the analytical derivation of complex cell properties from the slowness principle presented in [38], [41]. Sprekeler and Wiskott predict the formation of quadrature filter pairs to ensure slowly varying output signals on training input that is derived from applying translation to a static input image. At least locally, the mere translation of a wave image seems a reasonable first-order approximation of the simulated retinal waves. On the receptive field level only a part of the traveling wave front is visible in most cases, because the simulated waves are usually larger than the receptive field. This is of course not true in those cases where a wave emerges or decays within a receptive field, changes its size and shape, or, for example, the wave itself is smaller than the receptive field. Such cases also occur frequently and thereby provide an explanation for the fact that not all of the first 50 SFA units can be characterized as being a weighted superposition of quadrature filter pairs. Test simulations in which SFA units were trained with purely translation-based input stimuli show that all of the first 50 units indeed have this quadrature filter pair property.

The orientation selectivity exhibited by the SFA units obtained in the present study is well above experimental findings when measured with the orientation selectivity index OSI [13]. The reasons for such high OSI values have been investigated and presented in the Results section. The fact that orientation selectivity is overestimated, however, does not imply that the slowness hypothesis has to be dismissed. An additional objective may be necessary to capture all properties of complex cells. Perhaps a different preprocessing of the SFA units' responses needs to be done (e.g. rectification) before computing the OSI. Neural network implementations of the slowness principle have been applied in other studies [8], [27] and a neural implementation of SFA has been proposed in [63]. When trained with retinal wave image sequences, those implementations should also reproduce the phase invariance property. It would be interesting to explore how well these implementations fare in accounting for other complex cell properties, compared to the SFA algorithm used in this study.

### Experimental predictions

To the best of our knowledge, there are no studies that describe the properties of complex cells shortly after or shorty before birth. The experimental findings cited in this work are primarily derived from simple cells. However, the results of our work predict that complex cells could already be present at the time of birth or shortly after, possibly in some preliminary form. It seems likely that this is the case at least in some animal species, such as horse or giraffe, because their offspring is born at a very advanced developmental stage. The freshly born foals or calves are able to stand and follow their mother within the first hour after birth. It seems likely that these animals, even at this young age, can recognize and differentiate objects (e.g. other members of their species) independently of their position in the visual field, possibly using also other sensory cues such as olfactory or auditory signals. Complex cells are a prime candidate for the basis of position invariant object recognition [32], [64] and therefore it is likely that the animal species mentioned are born with an, at least partially developed, complex cell system already in place. Thus it would be very interesting to identify complex cells in animals shortly after eye opening and compare their properties to complex cells of mature animals of the same species.

Given that our first prediction can be experimentally verified, i.e. if it becomes possible to record from complex cells in animal species before or shortly after the onset vision, then a second, more principled, prediction concerning the emergence of simple and complex cells can be derived. The performed control experiments demonstrate the dependence of the SFA units on the transformations that are present in the training stimuli. If one could disrupt the temporal structure of retinal waves while leaving the spatial properties intact, then, according to the theory behind SFA, the development of complex cells should be impaired. The development of simple cells, on the other hand, should not be hindered, because the theory of sparse coding is based on spatial statistics only, making no reference to the temporal properties of the training data. Currently the necessary technology may not be available to actually perform such an experiment. However, it is already possible to pharmacologically block natural retinal waves [65] and further advances in the development of large-scale high-density multielectrode arrays [66] and retinal implants [67] may make it possible to artificially induce spatially coherent bursting patterns at the level of the retina. If such a setup becomes available, replaying recorded natural retinal wave images in a randomized order (by shuffling the frames) should impair development of complex cells stronger than the development of simple cells.

### Limitations

The results presented in this study can only give a rather simplified account of the processes that come together in structuring the neural circuitry of sensory systems. Specifically, our study addresses only a particular part of the development of the early visual system, namely complex cells. Already the clear-cut distinction between simple and complex cells is most likely too sharp, as it seems more likely to be a continuum between purely simple and purely complex cells [68]. Furthermore, in many species endogenously generated wave-like activity patterns are not only present in the retina but throughout the entire developing neural system, before and after birth [18]. It is likely that there are factors influencing the natural development that were not considered in this study.

With the present study we aim at showing how far one can get from two simple computational principles (endogenous activity and the slowness coding objective). Our results indicate that a number of interesting properties can emerge from these simple ingredients already, yet they cannot account for the true complexity of our intricate neural system.

### Conclusion

In conclusion we find that our simulation results support the hypothesis that the slowness objective, here manifested by SFA, is compatible with an innate learning mechanism that learns on endogenous activity in the same manner as on actual visual input. A large portion of the SFA units obtained from training with retinal wave image sequences share relevant properties with cortical complex-cells. Thereby we provide a theoretical account for the emergence of complex-cells prior to eye opening that allows for further refinement by exposure to natural visual input.

## Supporting Information

Text S1 In this Supporting Information we provide the analytical derivation of a formula for the orientation selectivity index (OSI) of a linear combination of Gabor wavelet quadrature filter pairs (Gabor-QFPs). The derived formula is the basis for sampling OSI values from the Gabor-QFP model and generating the OSI distribution shown in Figure 8 **C**.
(PDF)
