# Supplementary material for: Slow Feature Analysis on Retinal Waves Leads to V1 Complex Cells
Source: PLoS Comput Biol. 2014 May 8;10(5):e1003564. doi: 10.1371/journal.pcbi.1003564 (PMC4014395; doi:10.1371/journal.pcbi.1003564)
Supplement: Text S1 — In this Supporting Information we provide the analytical derivation of a formula for the orientation selectivity index (OSI) of a linear combination of Gabor wavelet quadrature filter pairs (Gabor-QFPs). The derived formula is the basis for sampling OSI values from the Gabor-QFP model and generating the OSI distribution shown in Figure 8 C . (PDF) [file pcbi.1003564.s001.pdf]

Slow Feature Analysis on Retinal Waves Leads to V1 Complex Cells – Supporting Information S1 –

Sven Dähne*[1,4], Niko Wilbert[2,4], Laurenz Wiskott[2,3,4]

[1]Machine Learning Group, Department of Computer Science, Berlin Institute of Technology
[2]Institute for Theoretical Biology, Humboldt-University Berlin
[3]Institute for Neuroinformatics, Ruhr-University Bochum
[4]Bernstein Center for Computational Neuroscience, Berlin

1 Gabor Quadrature Filter Pair Model

In this Section we derive a formula for the orientation selectivity index (OSI) [1] of a linear combination of Gabor wavelet quadrature filter pairs (Gabor-QFPs).

The orientation tuning curve of the ith Gabor-QFP, here denoted by $t_i(\phi)$, is a one dimensional function of the input grating's orientation angle. The curve has two maxima, one at $\phi_i^* < \pi$ and one at $\phi_i^{**} = \phi_i^* + \pi$, and it is obtained by a circular section through the amplitude spectrum of one of the Gabor filters at the preferred frequency f^*. Both filters of a quadrature filter pair have the same Fourier amplitude spectrum. They only differ in their phase spectrum. By evaluating the amplitude spectrum in polar coordinates, i.e. as a function of frequency f and orientation ϕ, here denoted by $A(f, \phi)$, the tuning curve is given by $t_i(\phi) = A_i(f^*, \phi)$.

The Fourier amplitude spectrum of a 2d Gabor function is equal to the sum of two Gaussians in Fourier space, centered at the points (f^*, ϕ^*) and (f^*, ϕ^{**}). The width of the Gaussians in Fourier space σ_f is inversely proportional to the width of the Gaussian envelope of the Gabor function in image space σ_x, i.e. $\sigma_f = \frac{1}{2\pi\sigma_x}$. The curved section through the 2d Gaussians in Fourier space can be approximated by a straight line section, which is a 1d Gaussian of width σ_f. Thus, $t_i(\phi)$ is modeled with two one-dimensional Gaussians, centered at ϕ_i^* and ϕ_i^{**}, respectively.

Recall the definition of the OSI: $100 * \frac{F2}{F2+F0} = \frac{100}{1+\frac{F0}{F2}}$, with $F0$ being the mean value and $F2$ the amplitude of the second harmonic. Because the Gabor-QFP tuning curve is a periodic function with a period of π radians (or 180°), it is equivalent to consider

*sven.daehne@bccn-berlin.de

$t_i(\phi)$ only in the range from 0 to π and to compute $F0$ and $F1$ of $t_i(\phi)$ confined to this interval.

The tuning curve of a linear combination of Gabor-QFP is the linear combination of the individual tuning curves, i.e. $t(\phi) = \sum_i^N w_i t_i(\phi)$. In order to compute the OSI for $t(\phi)$, an expression for its complex Fourier spectrum, $\mathcal{F}_t(f)$ is required. Once the spectrum is obtained, the amplitudes are given by the absolute value of the complex Fourier spectrum, e.g $F0 = |\mathcal{F}_t(0)|$ and $F1 = |\mathcal{F}_t(1)|$ with $|\mathcal{F}_t| = \sqrt{\mathcal{F}_t \overline{\mathcal{F}}_t}$.

First of all, the linearity of the Fourier transform is applied to yield an expression for $\mathcal{F}_t$ in terms of the Fourier transforms of the individual tuning curves $\mathcal{F}_{t_i}$, and the respective weights w_i

$$\mathcal{F}_t = \sum_i^N w_i \mathcal{F}_{t_i}. \tag{1}$$

Because all $t_i(\phi)$ are shifted versions of each other, each t_i can be expressed as the result of the convolution of a Gaussian, centered at 0°, with a shifted Dirac δ function, i.e. $t_i(\phi) = t_0(\phi) * \delta_i(\phi)$, where $*$ denotes convolution and $\delta_i(\phi) = \delta(\phi - \phi_i^*)$. This simplifies equation (1), because we can apply the convolution theorem, which expresses the Fourier transform of a convolution of two functions as the product of the Fourier transformed functions. $\mathcal{F}_{t_i}$ therefore becomes $\mathcal{F}_{t_0} \cdot \mathcal{F}_{\delta_i}$, which decomposes $\mathcal{F}_t$ further into

$$\mathcal{F}_t = \sum_i^N w_i \mathcal{F}_{t_i} = \sum_i^N w_i \mathcal{F}_{t_0} \mathcal{F}_{\delta_i} = \mathcal{F}_{t_0} \sum_i^N w_i \mathcal{F}_{\delta_i}. \tag{2}$$

Now an expression for $|\mathcal{F}_t|$ in terms of $\mathcal{F}_{t_0}$ and $\mathcal{F}_{\delta_i}$ can be formulated:

$$|\mathcal{F}_t| = \sqrt{\mathcal{F}_t \overline{\mathcal{F}}_t} = \sqrt{\mathcal{F}_{t_0} \sum_i^N w_i \mathcal{F}_{\delta_i} \overline{\mathcal{F}_{t_0} \sum_j^N w_j \mathcal{F}_{\delta_j}}} = \sqrt{\mathcal{F}_{t_0} \overline{\mathcal{F}_{t_0}} \sum_{i,j}^N w_i w_j \mathcal{F}_{\delta_i} \overline{\mathcal{F}_{\delta_j}}}. \tag{3}$$

In order to plug in actual values, expressions for $\mathcal{F}_{t_0}$ and $\mathcal{F}_{\delta_i}$ are given next. The zero-centered tuning curve, normalized to unit amplitude, is given by $t_0(\phi) = \exp\left(-\frac{1}{2}(\frac{\phi}{\sigma_\phi})^2\right)$, with $\sigma_\phi = \frac{\sigma_f}{\pi f^*} = \frac{1}{2\pi^2 \sigma_x f^*}$. Note that σ_ϕ is given as a fraction of 1, not in radians or degrees. Likewise, σ_x is specified in units of image side length, not in pixels. The Fourier transform of $t_0(\phi)$ is also a Gaussian:

$$\mathcal{F}_{t_0}(f) = \overline{\mathcal{F}_{t_0}(f)} = \sqrt{2\pi\sigma_\phi^2} \exp\left(-\frac{1}{2}\left(2\pi\sigma_\phi f\right)^2\right). \tag{4}$$

The Fourier transform of $\mathcal{F}_{\delta_i}$ is a complex sinusoid in frequency space:

$$\mathcal{F}_{\delta_i}(f) = \exp\left(-\mathrm{i}2\phi_i^* f\right). \tag{5}$$

Equations 4 and 5 can now be substituted into equation 3:

$$|\mathcal{F}_t(f)| = 2\pi\sigma_\phi^2 \exp\left(-\left(2\pi\sigma_\phi f\right)^2\right) \sum_{i,j}^N w_i w_j \exp\left(\mathrm{i}2f\left(\phi_j^* - \phi_i^*\right)\right) \tag{6}$$

$$= 2\pi\sigma_\phi^2 \exp\left(-\left(2\pi\sigma_\phi f\right)^2\right) \sum_{i,j}^N w_i w_j \cos\left(2f\left(\phi_j^* - \phi_i^*\right)\right). \tag{7}$$

The complex exponential in the sum simplifies to a cosine, because summand (i,j) is the complex conjugate of summand (j,i), canceling out the imaginary part. All that is left now, is to evaluate the ratio of $|\mathcal{F}_t(f)|$ for $f = 0$ and $f = 1$ (since we consider the half interval 0 to π only), and to substitute this ratio in the OSI definition. By using equation (7) and the definition of the OSI, we have

$$
\begin{aligned}
\mathrm{OSI}_{\mathrm{G-QFP}} &= \frac{100}{1+\frac{F0}{F1}} && (8)\\
&= \frac{100}{1+\frac{|\mathcal{F}_t(0)|}{|\mathcal{F}_t(1)|}} && (9)\\
&= \frac{100}{1+\exp\left(2\pi^2\sigma_\phi^2\right)\sqrt{\frac{\sum_{i,j}^N w_i w_j}{\sum_{i,j}^N w_i w_j \cos\left(2\left(\phi_j^*-\phi_i^*\right)\right)}}} && (10)\\
&= \frac{100}{1+\exp\left(\frac{1}{2(\pi\sigma_x f^*)^2}\right)\sqrt{\frac{\sum_{i,j}^N w_i w_j}{\sum_{i,j}^N w_i w_j \cos\left(2\left(\phi_j^*-\phi_i^*\right)\right)}}} && (11)
\end{aligned}
$$

Equation (11) gives the orientation selectivity index of a linear combination of Gabor quadrature filter pairs in terms of their respective weights w_i and the parameters σ_x and f^*.

References

[1] B. Chapman and M. P. Stryker. Development of orientation selectivity in ferret visual cortex and effects of deprivation. *Journal of Neuroscience*, 13(12):5251–5262, December 1993.
